# Supplementary material for: Validation of aerial photogrammetry methods to measure body size, condition and mass in small cetaceans
Source: J Physiol. 2026 Jan 29;604(5):1788–808. doi: 10.1113/JP290419 (PMC12953016; doi:10.1113/JP290419)
Supplement: Supplementary file 2 — Fig. S1 Straight‐line measurements at six defined body positions (BL = body length from tip of rostrum to the end of the tail notch, AP = immediately anterior to pectoral fin, AD = immediately anterior to dorsal fin, PD = immediately posterior to dorsal fin, MG = mid‐genital and MP = mid‐peduncle). Fig. S2 Linear relationship between absolute body condition index (BCIabs) and standard body condition index (BCIstd) for bottlenose dolphins (intercept = 0.0057 ± 0.084, slope = 0.87 ± 0.073). N = 25. Table S1 Average height:width (HW) ratio of bottlenose dolphins for all measurement sites from 5% to 85% body length (BL) from the rostrum. Table S2 Parameter estimates from a linear mixed‐effect model of height:width (HW) ratio in bottlenose dolphins (Tursiops spp.) Table S3 Summary of demographic and body morphometric data for the measured bottlenose dolphins, including species, sex, age class, body length (BL), observed body condition index (BCIobs), body mass (BM) and body volume (BV). Table S4 Parameter estimates from a generalized linear mixed‐effects model (GLMM) of body length (BL) and width (W) in bottlenose dolphins (Tursiops spp.). [file TJP-604-1788-s002.docx]

## SUPPORTING INFORMATION

**Table S1**. Average height:width (HW) ratio of bottlenose dolphins for all measurement sites from 5% to 85% body length (BL) from the rostrum.

| HW Ratio | Measurement site (%BL from rostrum) | | | | | | | | | | | | | | | | |
| --- | --- | --- | --- | --- | --- | --- | --- | --- | --- | --- | --- | --- | --- | --- | --- | --- | --- |
|  | **5** | **10** | **15** | **20** | **25** | **30** | **35** | **40** | **45** | **50** | **55** | **60** | **65** | **70** | **75** | **80** | **85** |
| Average | 1.33 | 1.27 | 1.11 | 1.14 | 1.14 | 1.16 | 1.22 | 1.31 | 1.44 | 1.50 | 1.56 | 1.59 | 1.59 | 1.66 | 1.91 | 2.48 | 3.53 |
| Cliffy | 1.61 | 1.24 | 1.13 | 1.15 | 1.12 | 1.16 | 1.19 | 1.33 | 1.46 | 1.43 | 1.54 | 1.54 | 1.55 | 1.68 | 1.94 | 2.23 | 2.95 |
| Cohen | 1.18 | 1.33 | 1.17 | 1.13 | 1.16 | 1.19 | 1.28 | 1.38 | 1.51 | 1.54 | 1.58 | 1.51 | 1.58 | 1.71 | 1.93 | 2.48 | 3.68 |
| Dougy | 1.19 | 1.36 | 1.09 | 1.19 | 1.11 | 1.10 | 1.16 | 1.21 | 1.35 | 1.41 | 1.44 | 1.44 | 1.39 | 1.50 | 1.79 | 2.39 | 3.35 |
| Evie | 1.13 | 1.17 | 1.16 | 1.15 | 1.16 | 1.14 | 1.17 | 1.22 | 1.37 | 1.41 | 1.49 | 1.47 | 1.45 | 1.48 | 1.62 | 2.01 | 3.27 |
| Gemma | 1.31 | 1.39 | 1.20 | 1.24 | 1.18 | 1.25 | 1.27 | 1.41 | 1.51 | 1.50 | 1.57 | 1.63 | 1.63 | 1.67 | 2.03 | 2.90 | 4.98 |
| Hallie | 1.60 | 1.33 | 1.08 | 1.11 | 1.12 | 1.17 | 1.28 | 1.31 | 1.43 | 1.46 | 1.52 | 1.70 | 1.60 | 1.70 | 1.94 | 3.12 | 2.90 |
| Howie | 1.15 | 1.11 | 1.06 | 1.11 | 1.11 | 1.06 | 1.13 | 1.27 | 1.44 | 1.55 | 1.65 | 1.68 | 1.60 | 1.76 | 2.07 | 2.49 | 3.47 |
| Huey | 1.36 | 1.21 | 1.06 | 1.15 | 1.17 | 1.18 | 1.21 | 1.30 | 1.44 | 1.53 | 1.63 | 1.76 | 1.85 | 1.88 | 2.11 | 2.67 | 3.72 |
| Jinx | 1.69 | 1.33 | 1.19 | 1.14 | 1.12 | 1.14 | 1.21 | 1.27 | 1.41 | 1.45 | 1.49 | 1.48 | 1.51 | 1.62 | 1.89 | 2.60 | 3.80 |
| Kiama | 1.43 | 1.30 | 1.17 | 1.19 | 1.15 | 1.19 | 1.21 | 1.32 | 1.50 | 1.57 | 1.64 | 1.66 | 1.71 | 1.79 | 1.99 | 2.72 | 4.28 |
| Kyra | 1.43 | 1.26 | 1.08 | 1.11 | 1.14 | 1.20 | 1.29 | 1.40 | 1.55 | 1.61 | 1.66 | 1.72 | 1.75 | 1.73 | 1.98 | 2.61 | 3.87 |
| Melody | 1.38 | 1.27 | 1.06 | 1.16 | 1.18 | 1.13 | 1.13 | 1.29 | 1.54 | 1.71 | 1.84 | 1.83 | 1.76 | 1.72 | 1.81 | 2.29 | 3.23 |
| Moki | 1.05 | 1.19 | 1.09 | 1.15 | 1.14 | 1.16 | 1.19 | 1.28 | 1.38 | 1.39 | 1.49 | 1.52 | 1.52 | 1.52 | 1.90 | 2.37 | 3.60 |
| Nudge | 1.37 | 1.30 | 1.07 | 1.17 | 1.15 | 1.17 | 1.22 | 1.31 | 1.38 | 1.47 | 1.53 | 1.58 | 1.60 | 1.63 | 1.76 | 2.17 | 2.62 |
| RB | 1.09 | 1.20 | 1.09 | 1.11 | 1.08 | 1.10 | 1.24 | 1.36 | 1.51 | 1.66 | 1.80 | 1.83 | 1.86 | 1.83 | 2.01 | 2.53 | 3.44 |
| Raaf | 1.17 | 1.40 | 1.14 | 1.17 | 1.18 | 1.20 | 1.22 | 1.27 | 1.43 | 1.44 | 1.48 | 1.52 | 1.49 | 1.59 | 1.79 | 2.13 | 2.98 |
| Scooter | 1.15 | 1.27 | 1.13 | 1.10 | 1.13 | 1.20 | 1.28 | 1.40 | 1.51 | 1.59 | 1.64 | 1.54 | 1.54 | 1.64 | 1.95 | 2.56 | 3.64 |
| Sirius | 1.21 | 1.27 | 1.16 | 1.16 | 1.18 | 1.24 | 1.32 | 1.41 | 1.51 | 1.61 | 1.61 | 1.56 | 1.57 | 1.76 | 1.99 | 2.63 | 3.85 |
| Squeak | 1.21 | 1.36 | 1.13 | 1.07 | 1.11 | 1.15 | 1.21 | 1.33 | 1.43 | 1.52 | 1.56 | 1.66 | 1.65 | 1.68 | 1.99 | 2.51 | 3.50 |
| Starbuck | 1.50 | 1.37 | 1.15 | 1.18 | 1.17 | 1.20 | 1.24 | 1.34 | 1.45 | 1.45 | 1.50 | 1.51 | 1.48 | 1.45 | 1.78 | 2.23 | 3.31 |
| Stella | 1.42 | 1.37 | 1.12 | 1.15 | 1.12 | 1.09 | 1.10 | 1.15 | 1.21 | 1.30 | 1.37 | 1.37 | 1.44 | 1.65 | 1.83 | 2.10 | 2.36 |
| Sunnie | 1.25 | 1.34 | 1.12 | 1.10 | 1.07 | 1.11 | 1.19 | 1.29 | 1.47 | 1.49 | 1.57 | 1.62 | 1.64 | 1.79 | 2.06 | 2.72 | 4.15 |
| Tallula | 1.85 | 1.12 | 1.03 | 1.09 | 1.12 | 1.17 | 1.25 | 1.33 | 1.41 | 1.49 | 1.53 | 1.50 | 1.59 | 1.69 | 1.97 | 2.48 | 3.63 |
| Zac | 1.12 | 1.05 | 1.02 | 1.11 | 1.17 | 1.22 | 1.30 | 1.38 | 1.46 | 1.46 | 1.45 | 1.44 | 1.48 | 1.48 | 1.91 | 2.64 | 4.02 |
| Zippa | 1.33 | 1.27 | 1.07 | 1.09 | 1.13 | 1.19 | 1.23 | 1.28 | 1.44 | 1.52 | 1.53 | 1.60 | 1.55 | 1.55 | 1.84 | 2.38 | 3.62 |

**Table S2.** Parameter estimates from a linear mixed-effect model of height:width (HW) ratio in bottlenose dolphins (*Tursiops* spp.)

| **Effect Type** | **Term** | **Estimate** | **Std. Error** | **t value** |
| --- | --- | --- | --- | --- |
|  |  |  |  |  |
| **Fixed** | (Intercept) | 2.324 | 0.562 | 4.133 |
|  | BL (centered) | -0.200 | 0.237 | -0.842 |
|  | W at 40%BL | -3.425 | 3.304 | -1.037 |
|  | Species (*T.truncatus*) | 0.038 | 0.050 | 0.763 |
|  | Sex | -0.036 | 0.056 | -0.645 |

**Table S3**. Summary of demographic and body morphometric data for the measured bottlenose dolphins, including species, sex, age class, body length (BL), observed body condition index (BCI_obs_), body mass (BM), and body volume (BV).

| Dolphin ID | Species | Sex | Age | Age class | BL (m) | BCI_obs_ | BM (kg) | BV (m^3^) | BD (kg/ m^3^) |
| --- | --- | --- | --- | --- | --- | --- | --- | --- | --- |
| Jinx | *T. truncatus* | female | 17 | adult | 2.75 | -0.029 | 224 | 0.240 | 933 |
| Evie | *T. truncatus* | female | 14 | adult | 2.58 | -0.114 | 174 | 0.186 | 937 |
| Gemma | *T. truncatus* | female | 34 | adult | 2.42 | 0.267 | 205 | 0.225 | 912 |
| Moki | *T. truncatus* | female | 44 | adult | 2.77 | 0.080 | 274 | 0.272 | 1007 |
| Squeak | *T. truncatus* | female | 44 | adult | 2.57 | 0.123 | 167 | 0.233 | 983 |
| Sunnie | *T. truncatus* | female | 20 | adult | 2.67 | -0.002 | 223 | 0.228 | 976 |
| Scooter | *T. truncatus* | female | 34 | adult | 2.55 | 0.220 | 240 | 0.248 | 968 |
| Stella | *T. truncatus* | female | 9 | juvenile | 2.49 | 0.070 | 193 | 0.204 | 944 |
| Starbuck | *T. truncatus* | male | 24 | adult | 2.99 | -0.033 | 283 | 0.297 | 953 |
| Zac | *T. truncatus* | male | 30 | adult | 2.60 | -0.041 | 201 | 0.205 | 980 |
| Sirius | *T. truncatus* | male | 44 | adult | 2.73 | -0.026 | 234 | 0.236 | 991 |
| Kiama | *T. truncatus* | male | 19 | adult | 2.73 | 0.078 | 245 | 0.261 | 937 |
| Cohen | *T. truncatus* | male | 27 | adult | 2.80 | -0.186 | 251 | 0.211 | 1191 |
| Dougy | *T. truncatus* | male | 7 | juvenile | 2.31 | 0.067 | 167 | 0.168 | 995 |
| Melody | *T. aduncus* | female | 13 | adult | 2.10 | -0.071 | 114 | 0.114 | 998 |
| Tallula | *T. aduncus* | female | 13 | adult | 2.24 | -0.074 | 139 | 0.135 | 1033 |
| Hallie | *T. aduncus* | female | 28 | adult | 2.17 | 0.125 | 157 | 0.151 | 1040 |
| Zippa | *T. aduncus* | female | 23 | adult | 2.18 | -0.066 | 130 | 0.126 | 1028 |
| Kyra | *T. aduncus* | female | 9 | juvenile | 1.96 | 0.027 | 113 | 0.105 | 1072 |
| Cliffy | *T. aduncus* | male | 17 | adult | 2.35 | -0.054 | 163 | 0.155 | 1048 |
| Howie | *T. aduncus* | male | 14 | adult | 2.32 | -0.133 | 154 | 0.138 | 1116 |
| Huey | *T. aduncus* | male | 16 | adult | 2.14 | -0.092 | 124 | 0.117 | 1058 |
| Nudge | *T. aduncus* | male | 16 | adult | 2.20 | 0.117 | 140 | 0.155 | 904 |
| RB | *T. aduncus* | male | 34 | adult | 2.23 | 0.006 | 158 | 0.144 | 1094 |
| Raaf | *T. aduncus* | male | 9 | juvenile | 2.31 | -0.117 | 122 | 0.139 | 879 |

**Table S4.** Parameter estimates from a generalized linear mixed-effects model (GLMM) of body length (BL) and width (W) in bottlenose dolphins (*Tursiops* spp.).

| **Effect Type** | **Term** | **Estimate** | **Std. Error** | **z value** | **p-value** |
| --- | --- | --- | --- | --- | --- |
| **Fixed** | Intercept | 0.2981 | 0.0097 | 30.65 | 2×10^-16^*** |
|  | UAV vs None | -0.00087 | 0.00188 | -0.46 | 0.6430 |
|  | Inspire 2 vs Mavic 3 | 0.00421 | 0.00277 | 1.52 | 0.1287 |
|  | Inspire 2: Stationary vs Swimming | 0.00288 | 0.00213 | 1.35 | 0.1765 |
|  | Mavic 3: Stationary vs Swimming | 0.00431 | 0.00073 | 5.89 | 3.86×10^-9^*** |
|  | Species (*T. truncatus*) | 0.0623 | 0.00756 | 8.23 | 2×10^-16^*** |
|  | Site: AP | -0.00919 | 0.00329 | -2.80 | 0.0051 |
|  | Site: MG | -0.1429 | 0.00347 | -41.13 | 2×10^-16^*** |
|  | Site: MP | -0.2291 | 0.00316 | -72.46 | 2×10^-16^*** |
|  | Site: PD | -0.0581 | 0.00314 | -18.52 | 2×10^-16^*** |
|  | Site: BL | 1.8897 | 0.01067 | 177.13 | 2×10^-16^*** |
|  | Species:*T.truncatus*:  SiteAP | -0.02099 | 0.00423 | -4.97 | 6.85×10^-7^*** |
|  | Species:*T.truncatus*:  SiteMG | -0.01519 | 0.00439 | -3.46 | 0.000054** |
|  | Species:*T.truncatus*:  SiteMP | -0.04497 | 0.00425 | -10.58 | 2×10^-16^*** |
|  | Species:*T.truncatus*:  SitePD | -0.00744 | 0.00405 | -1.84 | 0.06599 |
|  | Species:*T.truncatus*:  SiteBL | 0.3354 | 0.01812 | 18.51 | 2×10^-16^*** |

**Table 2.** Anova Chi-Square test for the generalized linear mix model examining the effects of UAV, species, body position, and the interaction between species and body position on bottlenose dolphin body length (BL), and width (W) measurements.

| **Predictor** | **Chi-Square** | **Df** | **p-value** |
| --- | --- | --- | --- |
| **UAV** | 39.743 | 4 | < 0.001 |
| **Species** | 43.116 | 1 | < 0.001 |
| **Body position** | 81084.885 | 5 | < 0.001 |
| **Species:Body position** | 536.621 | 5 | < 0.001 |


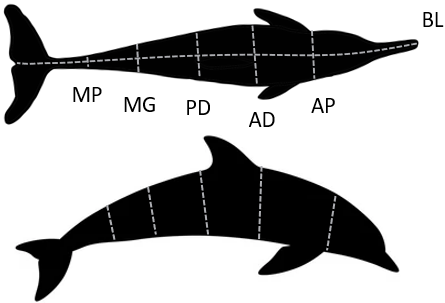


**Fig. S1**. **Straight-line measurements at 6 defined body positions** (BL = body length from tip of rostrum to the end of the tail notch, AP = immediately anterior to pectoral fin, AD = immediately anterior to dorsal fin, PD = immediately posterior to dorsal fin, MG = mid-genital, and MP = mid-peduncle).


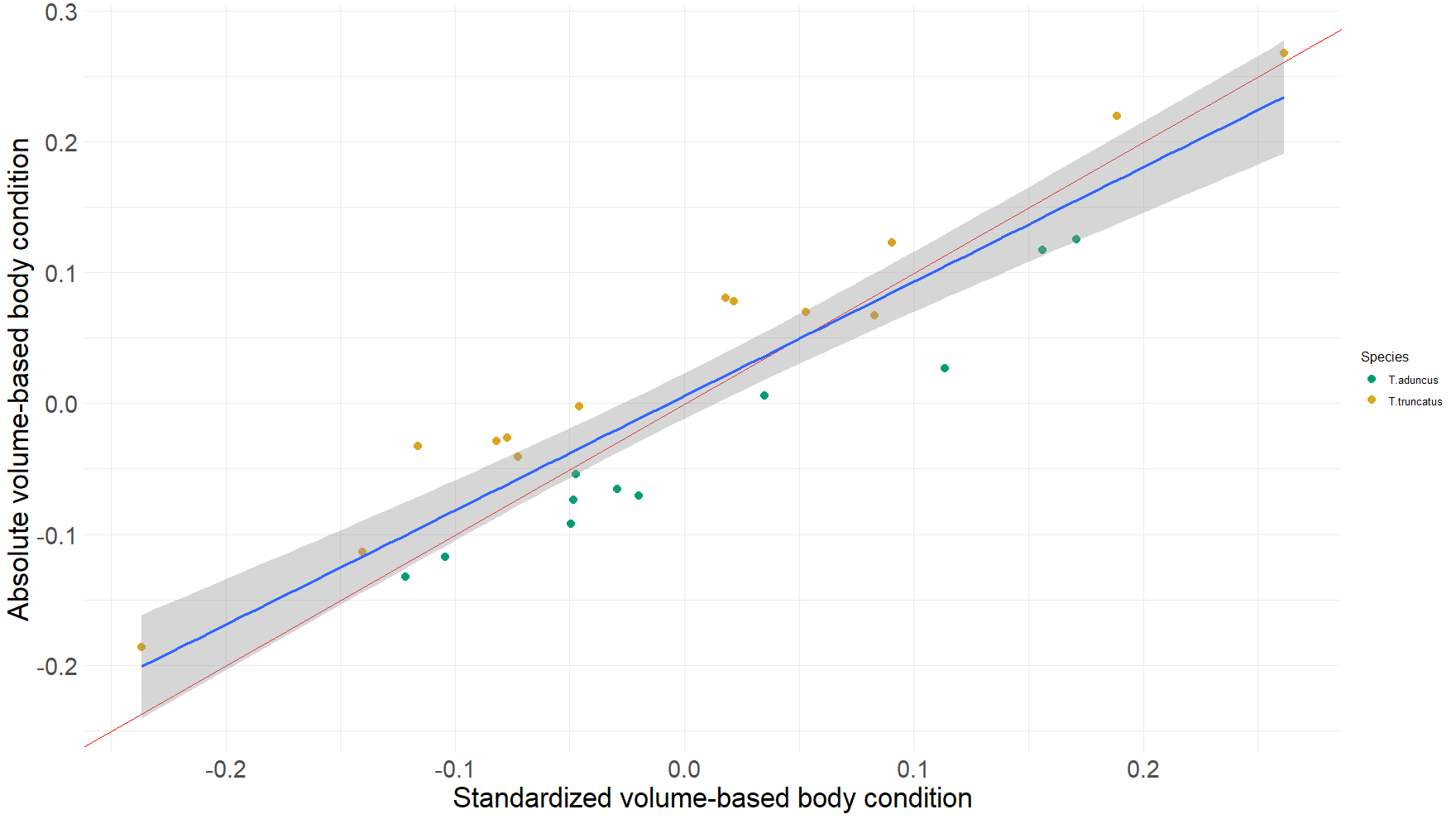


**Fig. S2:** Linear relationship between absolute body condition index (BCI_abs_) and standard body condition index (BCI_std_) for bottlenose dolphins (intercept = 0.0057 ± 0.084, slope = 0.87 ± 0.073). N = 25.
